# Nutritional risk factors in patients with nasopharyngeal carcinoma: a cross-sectional study

**DOI:** 10.3389/fnut.2024.1386361

**Published:** 2024-05-20

**Authors:** Pengpeng Wang, Xueling Huang, Li Xue, Jinlian Liao, Jieying Liu, Jiaxiang Yu, Ting Li

**Affiliations:** ^1^Nursing College of Guangxi Medical University, Nanning, Guangxi, China; ^2^Department of Nursing, The First Affiliated Hospital of Guangxi Medical University, Nanning, Guangxi, China; ^3^Faculty of Medicine and Health Sciences, Universiti Putra Malaysia, Serdang, Malaysia

**Keywords:** nasopharyngeal carcinoma, nutrition risk screening 2002, nutrition, nutritional risk, factor

## Abstract

**Background:**

Patients with nasopharyngeal carcinoma are notably susceptible to high nutritional risks. If not addressed, this susceptibility can lead to malnutrition, resulting in numerous adverse clinical outcomes. Despite the significance of this issue, there is limited comprehensive research on the topic.

**Objective:**

The objective of our study was to identify nutritional risk factors in patients with nasopharyngeal carcinoma.

**Methods:**

For this cross-sectional study, we recruited a total of 377 patients with nasopharyngeal carcinoma. The Nutritional Risk Screening 2002 tool was used to assess their nutritional risk. These patients were divided into a well-nourished group (*n* = 222) and a nutritional risk group (*n* = 155). Potential risk factors were screened out using univariate analysis (*p* < 0.1). These factors were subsequently analyzed with multivariate logistic regression analysis (*p* < 0.05) to identify the nutritional risk factors for these patients.

**Results:**

Our findings indicated that increasing age (OR = 1.085, 95%CI: 1.053–1.117, *p* < 0.001), high number of radiation treatments (OR = 1.103, 95%CI: 1.074–1.132, *p* < 0.001), low BMI (OR = 0.700, 95%CI: 0.618–0.793, *p* < 0.001), and low albumin levels (OR = 0.852, 95%CI: 0.789–0.921, *p* < 0.001) are significant nutritional risk factors in patients with nasopharyngeal carcinoma.

**Conclusion:**

Increasing age, high number of radiation treatments, low BMI, and low albumin levels are significant nutritional risk factors in patients with nasopharyngeal carcinoma.

## Introduction

Nasopharyngeal carcinoma (NPC) is an epithelial carcinoma, one of the most common malignancies within the nasopharynx, and a subset of head and neck cancers (1, 2). It originates from the mucosal lining of the nasopharynx and is linked with genetic factors, environmental influences, and Epstein–Barr virus infections (1, 2). The clinical signs and symptoms of NPC are categorized into four groups: (i) Nasopharyngeal tumor symptoms, such as nasal obstruction, epistaxis, and nasal discharge; (ii) Eustachian tube dysfunction symptoms, including otitis media and hearing loss; (iii) Symptoms from tumor extension towards the skull base, which may cause headaches, diplopia, facial pain, and numbness or paresthesia; and (iv) Palpable neck masses (3). Compared to other cancers, NPC is rare. About 129,000 new people were diagnosed with NPC in 2018, representing just 0.7% of all cancers diagnosed in 2018 (2). However, its geographical spread around the world is very uneven; over 70% of new patients are from East and Southeast Asia (4). NPC patients are at high nutritional risk due to the disease effects and anti-tumor treatment. A previous study showed that 85% (2,750/3232) of NPC patients were at high nutritional risk (5). Malnutrition may occur in NPC patients at nutritional risk if they do not get nutritional support on time. Malnutrition could weaken patients’ immune systems, prolong patients’ hospital stays, bring about adverse treatment effects, lead to treatment interruptions, and have negative consequences for the prognosis and quality of life (6, 7).

Early identification and intervention of nutritional risk in NPC patients can help reduce the incidence of malnutrition. Nutrition screening is defined as identifying individuals who are malnourished or at risk of malnutrition to determine whether a detailed nutrition assessment is indicated (8). Nutrition Risk Screening 2002 (NRS2002) is commonly used as an initial screening tool to identify potential nutritional risks (9, 10).

Despite the potential impact of nutritional risk on NPC patients’ prognosis and quality of life, there is limited research on identifying nutritional risk factors in this population. While several studies have investigated factors for malnutrition among NPC patients, the evaluation of these factors has not been comprehensive. For example, some studies have only assessed demographic data and failed to analyze critical blood indicators. Furthermore, although certain studies have analyzed blood biomarkers, their analysis was limited to previously validated indicators and lacked comprehensiveness. Additionally, the sample size in these studies was small, limiting their generalizability. To address the gaps in existing literature, this study utilized NRS 2002 to assess the nutritional risks in NPC patients. It performed a comprehensive analysis of demographic data, lifestyle habits, tumor stages, treatments, and blood indicators to identify nutritional risk factors in this population.

## Materials and methods

**Figure 1:**
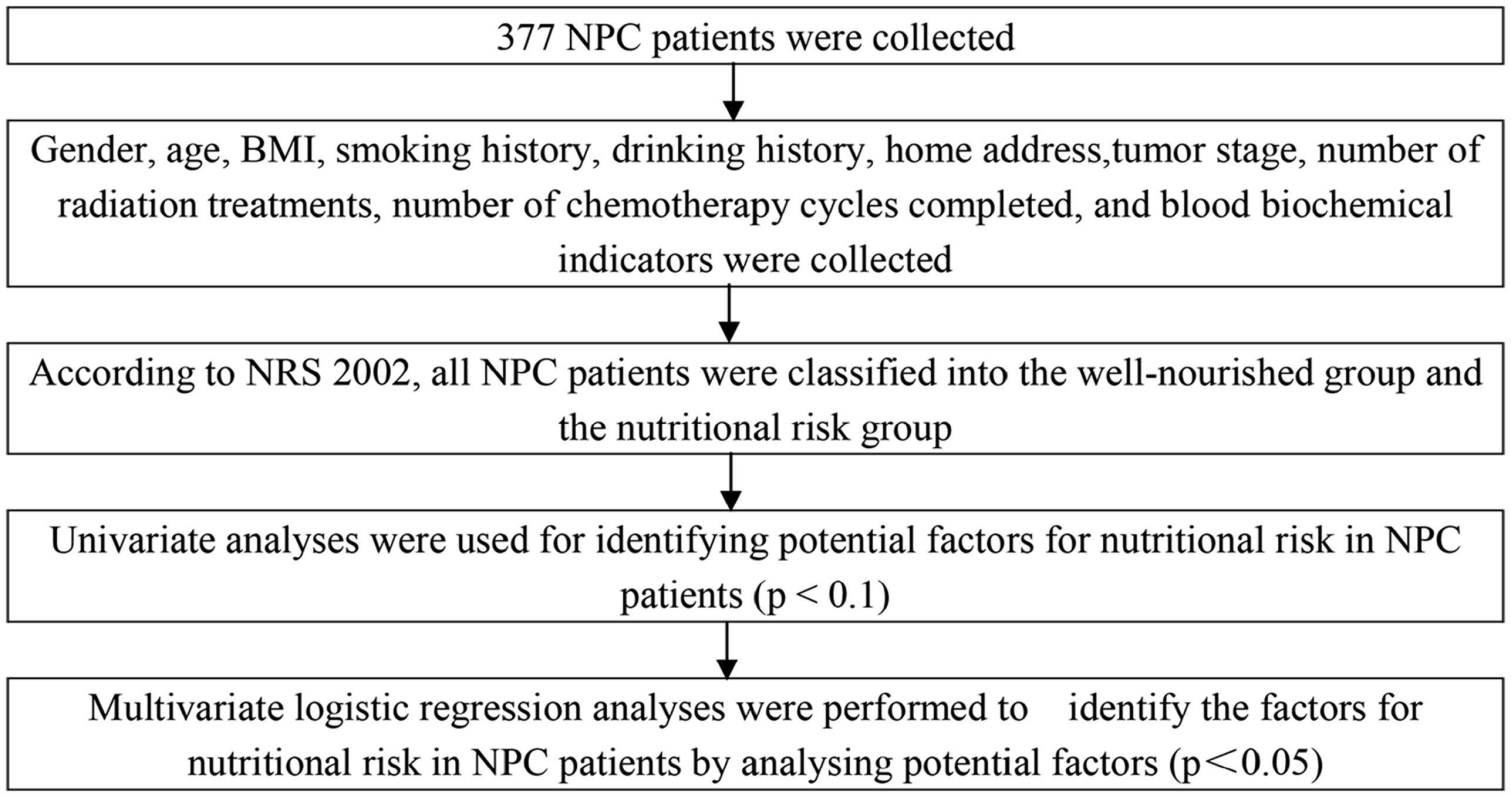
Study flow diagram.

The study flow diagram is presented in Figure 1.

### Patient selection

In this prospective cross-sectional study conducted from January 2022 to May 2023, we focused on NPC patients admitted to a general tertiary hospital. Utilizing a consecutive sampling approach, we selected all consecutively admitted NPC patients who met the inclusion criteria until our predetermined sample size was reached. The sample size was determined based on having at least 10 events for each predictor parameter. To ensure there was no duplication in data collection, meticulous checks were carried out using unique identifiers for each patient. All patients were informed, and informed consent was obtained. Based on the results of NRS 2002, we classified all NPC patients into two groups: the well-nourished group and the nutritional risk group. Demographic data, lifestyle habits, tumor stages, treatments, and blood indicators of NPC patients were analyzed to identify nutritional risk factors in this population.

### Inclusion and exclusion criteria

Inclusion criteria: (1) all newly diagnosed cases were confirmed by pathology; (2) no history of malignant tumors in other organs; and (3) age ≥ 18 years.

Exclusion criteria: NPC patients who had severe metabolic or nutritional diseases, as well as life-threatening illnesses, psychiatric disorders, or intellectual disabilities.

### Nutritional screening tool

NRS 2002 is widely used as a nutritional screening tool and was developed based on an analysis of 128 randomized clinical trials (10). The European Society for Parenteral and Enteral Nutrition guidelines recommend NRS 2002 for use in hospital settings as an effective and reliable nutritional screening tool (11). In addition, the Chinese Medical Association has recommended NRS 2002 to screen nutritional risk in hospitalized patients based on a report of 15,098 patients (12). Given its established reliability and effectiveness, we utilized NRS 2002 to screen the nutritional risk of NPC patients in this study.

**Figure 2:**
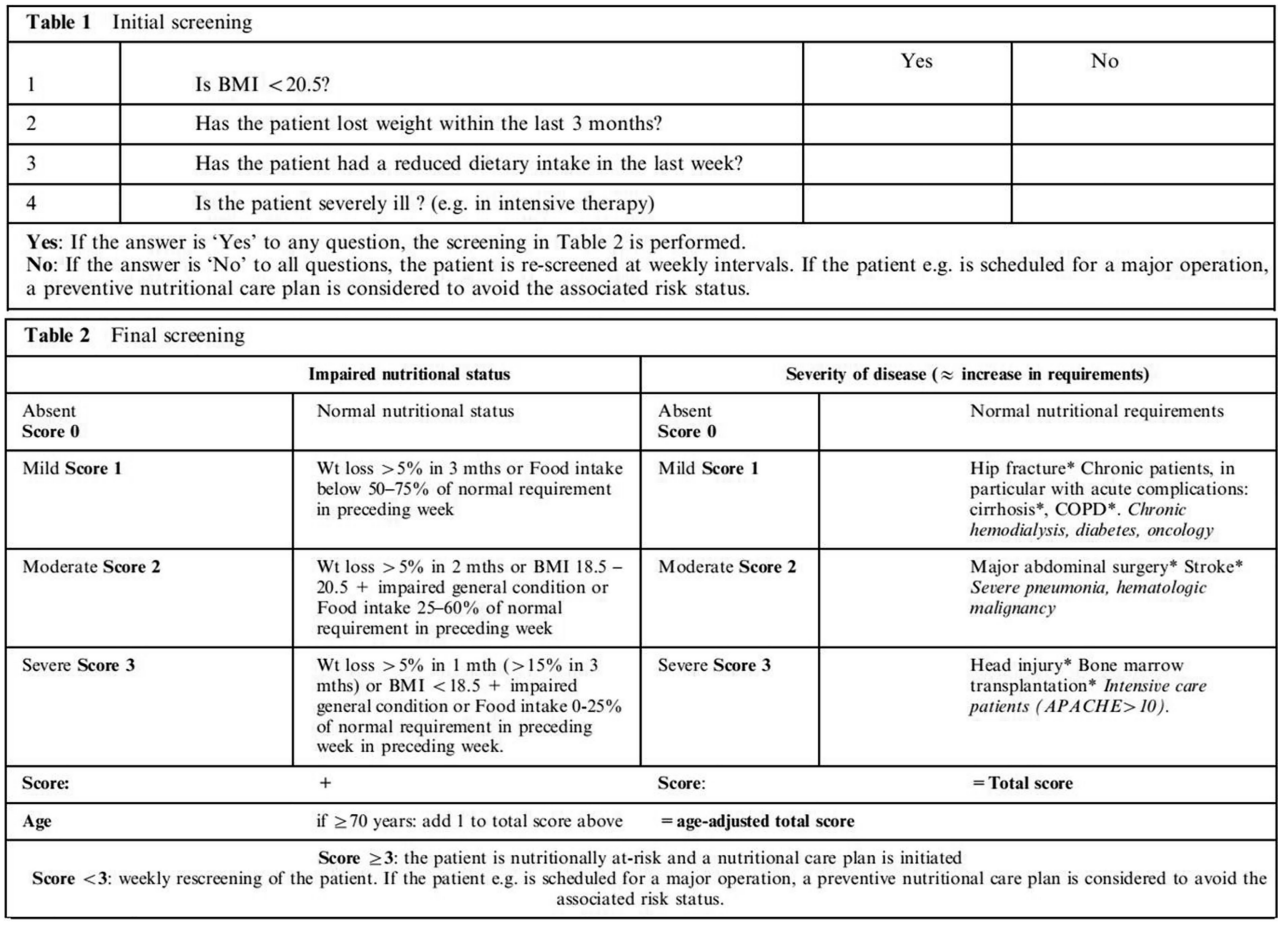
NRS 2002 (11). BMI, body mass index; Wt, weight; COPD, chronic obstructive pulmonary disease; APACHE, acute physiology and chronic health evaluation.

The NRS 2002 (Figure 2) consists of two components: initial screening and final screening. Initial screening includes four judgmental questions about BMI, weight loss, food intake, and severity of illness. If the patients answered “yes” to any of the four initial screening factors, they would proceed to the final screening. Otherwise, they are not currently at nutritional risk and do not need final screening. They need to be reviewed weekly for nutritional status. The final screening includes impaired nutritional status, severity of disease, and age scores, which are combined to screen nutritional risk. A total score ≥ 3 indicates a high nutritional risk (13). NPC patients were divided into a well-nourished group and a nutritional risk group, according to the NRS 2002.

### Data collection

All NPC patients in this hospital undergo routine blood biochemical tests before treatment to aid physicians in formulating appropriate treatment plans. Before undergoing the blood biochemical examination, each patient’s data including gender, age, body mass index (BMI), smoking history, drinking history, and home address, were collected face to face. Tumor stage, number of radiation treatments, number of chemotherapy cycles completed, and blood biochemical indicators were collected from the hospital information system. The NRS 2002 scores were collected by NRS 2002.

The World Health Organization classification criteria were used to classify the tumor stage (14). Smoking was defined as patients with a smoking history of >2 pack-years or current smoking. Drinking was defined as consuming alcohol at least once a week for more than a year, currently drinking, or having quit drinking for less than 3 years.

In this study, patients’ data were collected on admission. Concurrently, the NRS 2002 was employed to assess nutritional risk at admission. Expertly trained nurses, proficient in the NRS 2002, were responsible for all data collection, ensuring accuracy and uniformity in the screening process.

### Statistical analysis

All data were analyzed using SPSS26.0, and no data were missing. Medians with interquartile ranges [P25, P75] and means ± standard deviations (SD) were utilized to present quantitative data. First, a univariate analysis for each risk factor was performed to determine the potential risk factors. For the univariable analysis, we used the *t*-test, chi-squared, and Wilcoxon rank-sum tests for continuous, categorical, and graded or skewed distribution variables, respectively. Second, variables with *p* < 0.1 were carried forward to the logistic regression model, where we obtained ORs and 95% CIs. The significant variables with *p* values less than 0.05 were the nutritional risk factors in NPC patients. The investigators were trained uniformly. All data entry was double-checked.

### Statement of ethics

The study was approved by the Ethical Committee of the First Affiliated Hospital of Guangxi Medical University (approval number: NO. 2022-KT-Gui Wei-005).

## Results

The study included 377 patients, with 222 in the well-nourished group and 155 in the nutritional risk group. Among them, the mean age was 50.05 ± 12.09 years (range 20–84); 294 (78.0%) patients were <60 years old, and 83 (22.0%) were ≥ 60 years old; the mean BMI was 22.30 ± 3.34 kg/m^2^ (range 15.24–35.80 kg/m^2^); 44 (11.7%) had a BMI of <18.5 kg/m^2^, 74 (19.6%) had a BMI of 18.5–20.5 kg/m^2^, and 259 (68.7%) had a BMI of >20.5 kg/m^2^; 285 (75.6%) were male, and 92 (24.4%) were female; 194 (51.5%) had no history of smoking, while 183 (48.5%) had a current or previous history of smoking; 228 (60.5%) had no history of drinking, while 149 (39.5%) had a current or previous history of drinking; 257 (68.2%) lived in rural locations, 37 (9.8%) in suburban locations, and 83 (22%) in urban locations; 7 (1.9%) were in stage I, 25 (6.6%) were in stage II, 96 (25.5%) were in stage III, and 249 (66.0%) were in stage IV. The median values of the NRS 2002 scores in 377 patients were (P25: 1, P75: 4), with the overall range extending from 1 to 5. The data of 377 patients are shown in Table 1. The median values of the NRS 2002 scores in the well-nourished group were (P25: 1, P75: 1), with the overall range extending from 1 to 2. The median values of the NRS 2002 scores in the nutritional risk group were (P25: 3, P75: 4), with the overall range extending from 3 to 5.

**Table 1:** The data of 377 patients.

Variable	Category	*N*	%
Age (year)	<60	294	78
	≥60	83	22
Body mass index (kg/m^2^)	<18.5	44	11.7
	18.5–20.5	74	19.6
	>20.5	259	68.7
Gender	Male	285	75.6
	Female	92	24.4
Smoking	No	194	51.5
	Yes	183	48.5
Drinking	No	228	60.5
	Yes	149	39.5
Home address	Rural	257	68.2
	Suburban	37	9.8
	Urban	83	22
Tumor stage	I	7	1.9
	II	25	6.6
	III	96	25.5
	IV	249	66

Variable	Descriptive statistics	NPC patients (*N* = 377)
Age (year)	Mean ± SD	50.05 ± 12.09
	Range	20–84
Body mass index (kg/m^2^)	Mean ± SD	22.30 ± 3.34
	Range	15.24–35.80
The number of chemotherapy cycles completed	(P25, P75)	(1, 3)
	Range	0–7
The number of radiation treatments	(P25, P75)	(0, 20)
	Range	0–35
NRS 2002 scores	(P25, P75)	(1, 4)
	Range	1–5
Total bilirubin (umol/l)	Mean ± SD	10.10 ± 6.06
Direct bilirubin (umol/l)	Mean ± SD	3.01 ± 1.83
Indirect bilirubin (umol/l)	Mean ± SD	7.09 ± 4.54
Total protein (g/l)	Mean ± SD	70.83 ± 6.71
Albumin (g/l)	Mean ± SD	41.56 ± 4.77
Globulin (g/l)	Mean ± SD	31.63 ± 5.00
Gamma-glutamyl transpeptidase (u/l)	Mean ± SD	39.92 ± 34.64
Total bile acids (umol/l)	Mean ± SD	7.06 ± 12.55
Aspartate aminotransferase (u/l)	Mean ± SD	27.86 ± 27.68
Alanine aminotransferase (u/l)	Mean ± SD	22.54 ± 17.60
Alkaline phosphatase (u/l)	Mean ± SD	76.35 ± 22.33
Prealbumin (mg/l)	Mean ± SD	248.36 ± 68.34
Cholinesterase (u/l)	Mean ± SD	8143.83 ± 1889.73
UREA (mmol/l)	Mean ± SD	4.61 ± 1.61
Creatinine (umol/l)	Mean ± SD	72.79 ± 18.44
Uric acid (umol/l)	Mean ± SD	341.79 ± 99.72
HCO3 (mmol/l)	Mean ± SD	26.32 ± 4.32
Creatinine clearance rate (ml/min)	Mean ± SD	96.68 ± 22.53
Cystatin C (mg/l)	Mean ± SD	0.86 ± 0.41
Potassium (mmol/l)	Mean ± SD	3.99 ± 0.39
Sodium (mmol/l)	Mean ± SD	138.70 ± 3.07
Chlorine (mmol/l)	Mean ± SD	103.72 ± 3.77
Calcium (mmol/l)	Mean ± SD	2.28 ± 0.13
Magnesium (mmol/l)	Mean ± SD	0.78 ± 0.12
Phosphorus (mmol/l)	Mean ± SD	1.16 ± 0.42
Retinol binding protein (mg/l)	Mean ± SD	44.56 ± 18.28

A univariate analysis for each risk factor was performed to determine the potential risk factors. Variables with *p* < 0.1 were the potential risk factors. In univariate analysis, there were statistically significant differences in age, BMI, the number of chemotherapy cycles completed, the number of radiation treatments, albumin, gamma-glutamyl transpeptidase, total bile acids, aspartate aminotransferase, alanine aminotransferase, prealbumin, cholinesterase, uric acid, potassium, and retinol binding protein (*p* < 0.1). The results are shown in Table 2.

**Table 2:** The results of univariate analysis.

Variable	Category	Well-nourished group (*n* = 222)	Nutritional risk group (*n* = 155)	X^2^/Z	*p*
Gender	Male	164	121	0.869	0.351
	Female	58	34		
Smoking	No	115	79	0.025	0.873
	Yes	107	76		
Drinking	No	132	96	0.234	0.628
	Yes	90	59		
Home address^a^	Rural	144	113	−1.554	0.12
	Suburban	25	12		
	Urban	53	30		
Tumor stage^a^	I	6	1	−0.846	0.397
	II	17	8		
	III	55	41		
	IV	144	105		

Variable	Well-nourished group (*n* = 222)	Nutritional risk group (*n* = 155)	t/Z	*p*
Age (year)	45.70 ± 10.38	56.27 ± 11.66	−9.25	<0.001
BMI (kg/m^2^)	23.59 ± 2.99	20.47 ± 2.94	10.02	<0.001
The number of chemotherapy cycles completed^a^	2 (1, 3)	3 (2, 4)	−5.85	<0.001
The number of radiation treatments^a^	0 (0, 1)	20 (0, 30)	−9.61	<0.001
Total bilirubin (umol/l)	9.92 ± 6.12	10.35 ± 5.97	−0.68	0.498
Direct bilirubin (umol/l)	2.98 ± 1.87	3.07 ± 1.74	−0.46	0.649
Indirect bilirubin (umol/l)	6.95 ± 4.48	7.29 ± 4.65	−0.72	0.473
Total protein (g/l)	70.70 ± 6.59	71.01 ± 6.90	−0.45	0.656
Albumin (g/l)	43.31 ± 4.03	39.05 ± 4.63	9.49	<0.001
Globulin (g/l)	31.39 ± 4.91	31.97 ± 5.13	−1.10	0.272
Gamma-glutamyl transpeptidase (u/l)	44.57 ± 39.34	33.27 ± 25.17	3.40	0.001
Total bile acids (umol/l)	8.07 ± 14.48	5.62 ± 8.96	1.88	0.061
Aspartate aminotransferase (u/l)	30.43 ± 35.20	24.19 ± 8.35	2.54	0.012
Alanine aminotransferase (u/l)	24.86 ± 20.34	19.21 ± 12.24	3.37	0.001
Alkaline phosphatase (u/l)	77.49 ± 21.63	74.72 ± 23.26	1.19	0.237
Prealbumin (mg/l)	264.56 ± 68.17	228.03 ± 63.45	5.27	<0.001
Cholinesterase (u/l)	8576.00 ± 1852.25	7524.84 ± 1772.19	5.52	<0.001
UREA (mmol/l)	4.60 ± 1.46	4.63 ± 1.81	−0.17	0.862
Creatinine (umol/l)	71.69 ± 17.69	74.37 ± 19.43	−1.39	0.165
Uric acid (umol/l)	357.45 ± 100.38	319.37 ± 39.65	3.71	<0.001
HCO3 (mmol/l)	26.20 ± 4.95	26.48 ± 3.23	−0.63	0.532
Creatinine clearance rate (ml/min)	96.42 ± 22.07	97.06 ± 23.23	−0.27	0.789
Cystatin C (mg/l)	0.86 ± 0.43	0.86 ± 0.38	0.05	0.962
Potassium (mmol/l)	4.03 ± 0.37	3.93 ± 0.42	2.44	0.015
Sodium (mmol/l)	138.58 ± 3.21	138.17 ± 2.71	1.28	0.201
Chlorine (mmol/l)	102.95 ± 2.82	102.49 ± 3.56	1.33	0.184
Calcium (mmol/l)	2.28 ± 0.12	2.28 ± 0.14	−0.70	0.487
Magnesium (mmol/l)	0.79 ± 0.11	0.78 ± 0.13	1.01	0.312
Phosphorus (mmol/l)	1.34 ± 0.30	1.18 ± 0.55	−0.97	0.331
Retinol binding protein (mg/l)	46.96 ± 15.15	41.12 ± 21.60	3.09	0.002

a The Wilcoxon rank-sum test was used to compare the groups.

Variables with *p* < 0.1 were included in the multivariable logistic regression model. The significant variables with *p* values less than 0.05 were the nutritional risk factors in NPC patients. The logistic regression analysis results showed statistically significant differences in age, BMI, the number of radiation treatments, and albumin (*p* < 0.05). Specifically, for every one-unit increase in age, the odds of nutritional risk increased by a factor of 1.085 (OR = 1.085, 95%CI: 1.053–1.117, *p* < 0.001). On the other hand, for every one-unit increase in BMI, the odds of nutritional risk decreased by a factor of 0.700 (OR = 0.700, 95%CI: 0.618–0.793, *p* < 0.001). Furthermore, for every one-unit increase in the number of radiation treatments, the odds of nutritional risk increased by a factor of 1.103 (OR = 1.103, 95%CI: 1.074–1.132, *p* < 0.001), and for every one-unit decrease in albumin levels, the odds of nutritional risk increased by a factor of 0.852 (OR = 0.852, 95%CI: 0.789–0.921, *p* < 0.001). These findings suggested that increasing age, high number of radiation treatments, low BMI, and low albumin levels were nutritional risk factors in NPC patients. The results are shown in Table 3.

**Table 3:** The results of the multivariable logistic regression model.

Variable	B	SE.	Wald	Sig.	Exp(B)	95%CI for EXP(B)	
						Lower	Upper
Age (year)	0.081	0.015	28.756	<0.001	1.085	1.053	1.117
BMI (kg/m^2^)	−0.357	0.064	31.249	<0.001	0.700	0.618	0.793
The number of radiation treatments	0.098	0.013	53.094	<0.001	1.103	1.074	1.132
Albumin (g/l)	−0.160	0.040	16.242	<0.001	0.852	0.789	0.921

## Discussion

NPC patients have a high nutritional risk due to various factors, such as the disease itself and treatment-related toxicities. The failure to provide timely nutritional support to NPC patients at nutritional risk can lead to malnutrition, resulting in adverse consequences. Despite the importance of this issue, limited studies have explored the nutritional risk factors in NPC patients. Our study aimed to identify the nutritional risk factors in NPC patients using the NRS 2002 to address this gap. Our findings showed that increasing age, high number of radiation treatments, low BMI, and low albumin levels were significant nutritional risk factors in NPC patients.

### Age

Nutritional risk is more common in older adults (15), as aging may be accompanied by the accumulation of diseases and impairments, such as depressive symptoms, cognitive and physical decline, emotional variations (16), and poor oral health (17). Moreover, elderly patients may have problems such as gastrointestinal hypofunction, weakened digestion and absorption capabilities, reduced liver function, and a diminished ability to metabolize nutrients, often combined with underlying diseases (18, 19). All of these factors may directly influence the balance between nutritional needs and intake (16). Even with adequate nutrition and energy intake, altered nutrient needs, compromised nutrient metabolism, and drug-nutrient interactions may affect the nutritional status of older people (20).

Our findings suggested that advanced age (OR = 1.085, 95%CI: 1.053–1.117, *p* < 0.001) was a nutritional risk factor in NPC patients. In the well-nourished group, 7.2% (16/222) of NPC patients were ≥ 60 years old. However, in the nutritional risk group, 43.2% (67/155) of NPC patients were ≥ 60 years old.

### The number of radiation treatments

The loss of appetite in patients during the radiotherapy phase of cancer treatment may be caused by the side effects of the treatment. Loss of appetite in patients can lead to serious nutritional problems that adversely affect a patient’s disease prognosis, treatment outcomes, and quality of life (21). Radiotherapy can cause side effects that can lead to reduced food intake, loss of nutrients, changes in energy expenditure, and weight loss (22–24). Weight loss, mucositis, and reduced food intake occur in about 80% of patients receiving radiotherapy to the head and neck or esophagus (25).

Our findings suggested that the greater the number of radiation treatments, the greater the nutritional risk in NPC patients (OR = 1.103, 95%CI: 1.074–1.132, *p* < 0.001). In our study, among patients who underwent ≥10 cycles of radiotherapy, 14.0% (31/222) were in the well-nourished group, while 63.2% (98/155) were in the nutritional risk group.

### BMI

BMI = weight (kg)/height^2^ (m^2^). BMI is a measure of the nutritional status of adults. A low BMI indicates a high risk of inadequate nutritional intake. BMI is often used to aid in screening for nutritional risk using many nutritional screening or assessment tools. For example, the NRS 2002 incorporates pre-screening with four questions. “Is the BMI of the patient <20.5 kg/m^2^” is one of the four questions in the pre-screening. Screening is performed if one of the four questions is answered positively (26). BMI is also a standard screening parameter in Mini Nutritional Assessment (MNA). In addition, the MNA score was positively correlated with BMI (27). A phenotypic criteria (non-Asian: low BMI < 20 kg/m^2^ if <70 years or < 22 kg/m^2^ if >70 years; Asia: low BMI < 18.5 kg/m^2^ if <70 years or < 20 kg/m^2^ if >70 years) was included in the Global Leadership Initiative on Malnutrition (GLIM) (28). Furthermore, BMI < 18.5 kg/m2 was defined as malnutrition based on the consensus statement of the European Society of Clinical Nutrition and Metabolism (29).

Our study showed that the low BMI (OR = 0.700, 95%CI: 0.618–0.793, *p* < 0.001) was a nutritional risk factor in NPC patients. In our study, the BMI was 23.59 ± 2.99 kg/m^2^ in the well-nourished group and 20.47 ± 2.94 kg/m^2^ in the nutritional risk group.

### Albumin

Traditionally, albumin has been used as a nutritional marker to quantify the amount of plasma circulating protein and is thus considered to reflect nutritional status. Albumin <35 g/L indicates hypoalbuminemia and persistent hypoalbuminemia is an important indicator of malnutrition (30). Dietary protein intake (DPI) directly influences albumin concentrations, and inadequate DPI could lead to a decrease in the rate of albumin, which may have little impact on albumin levels in the short term. Although the decrease in the rate of albumin was small, it was clinically significant. Eckart et al. demonstrated that nutritional risk was associated with albumin in adult patients (31). In addition, albumin is a visceral protein that is a sensitive indicator of marginal nutrient deficiency and can reflect changes in protein-caloric nutrition. Albumin has been used as a marker of nutritional status in orthopedic patients (32). Prenner et al. demonstrated that albumin was an objective parameter that could provide time-effective and cost-controlled evidence regarding malnutrition in patients after heart transplantation.

Our study showed that the lower the albumin levels, the higher the nutritional risk in NPC patients (OR = 0.852, 95%CI: 0.789–0.921, *p* < 0.001). The reasons might be as follows: Firstly, treatment for NPC can induce acute inflammation, such as acute oral mucositis (33), dysphagia (34), and gastrointestinal reactions (35), which can affect nutritional intake. Secondly, the acute inflammations (33, 34, 36)caused by treatment for NPC may reduce albumin concentrations. In our study, patients in the nutritional risk group received more radiotherapy than those in the well-nourished group. Finally, the albumin level is reduced with the progression of NPC (37).

### Strengths and limitations

This study provides several notable advantages compared to previous research studies. Firstly, our research comprehensively analyzed various factors, such as demographic data, lifestyle habits, tumor stage, treatments, and blood indicators of NPC patients, to identify nutritional risk factors in this population. Secondly, our study had a large sample size, enhancing the findings’ validity and reliability. Thirdly, we used NRS2002 as the primary nutritional screening tool, a widely accepted method for identifying nutritional risk in hospitalized patients.

Despite its strengths, our study has limitations. The cross-sectional design prevents us from inferring causality between variables and nutritional risk in NPC patients. To draw causal conclusions, randomized controlled trials (RCTs) or longitudinal studies are required. Additionally, we did not examine the link between patient psychology and nutritional risk.

### The meaning of the study

Our study identifies nutritional risk factors in NPC patients, providing theoretical and practical guidance for clinical nutritional risk screening and support. Equipped with a deep understanding of factors linked to nutritional risks, nurses can educate patients more effectively, helping them grasp their risks and suggesting strategies for managing and mitigating these vulnerabilities. By collaborating closely with dieticians, nurses can develop tailored dietary and nutritional regimens that meet the daily needs of NPC patients and enhance their overall quality of life and treatment outcomes. Prompt identification of nutritional factors allows nursing professionals to intervene early, preventing further deterioration in patients’ nutritional health. This proactive approach not only prevents related complications but also can reduce the length of hospital stays, alleviating the burden on the healthcare system. In summation, our study offers valuable guidance for nursing and medical staff, ensuring enhanced care for NPC patients, improved nutritional health, and an overall better quality of life.

## Conclusion

Increasing age, high number of radiation treatments, low BMI, and low albumin levels were nutritional risk factors in NPC patients. These results may be useful in guiding clinical nutritional risk screening and interventions for this population. Further research is needed to confirm these findings and investigate additional nutritional risk factors in NPC patients.

## Data availability statement

The raw data supporting the conclusions of this article will be made available by the authors, without undue reservation.

## Ethics statement

The study was approved by the Ethical Committee of the First Affiliated Hospital of Guangxi Medical University (approval number: No. 2022-KT-Gui Wei-005). The studies were conducted in accordance with the local legislation and institutional requirements. The participants provided their written informed consent to participate in this study.

## Author contributions

PW: Conceptualization, Data curation, Funding acquisition, Investigation, Methodology, Software, Writing – original draft, Writing – review & editing. XH: Conceptualization, Investigation, Methodology, Visualization, Writing – original draft, Writing – review & editing. LX: Data curation, Formal analysis, Project administration, Resources, Supervision, Writing – original draft, Writing – review & editing. JinL: Formal analysis, Investigation, Project administration, Resources, Validation, Visualization, Writing – original draft, Writing – review & editing. JieL: Data curation, Methodology, Project administration, Software, Supervision, Writing – original draft, Writing – review & editing. JY: Data curation, Formal analysis, Investigation, Software, Writing – original draft, Writing – review & editing. TL: Data curation, Formal analysis, Methodology, Supervision, Validation, Writing – original draft, Writing – review & editing.
